# Using Perforated Liners to Combat the Detrimental Effects of Excessive Sweating in Lower Limb Prosthesis Users

**DOI:** 10.33137/cpoj.v3i2.34610

**Published:** 2020-09-03

**Authors:** K.C. Davies, M McGrath, Z Savage, A Stenson, D Moser, S Zahedi

**Affiliations:** 1 Blatchford Group, Unit D Antura, Bond Close, Basingstoke, RG24 8PZ, UK.; 2 Sheffield Mobility & Specialised Rehabilitation Centre, Northern General Hospital, Sheffield, UK.

**Keywords:** Prosthetics, Sweat, Prosthetic Liner, Perforated, Amputation, Residual limb health, Silicone liner, Amputee, Dysvascular, Temperature

## Abstract

**BACKGROUND:**

Excessive sweating of the residual limb has a substantial effect on the daily activities of people with lower limb amputation. Prosthetic liners offer protection and comfort to sensitive areas but often exacerbate perspiration. They act as insulators, trapping sweat on the skin’s surface to the detriment of skin health. Recently, liners with perforations have been developed, allowing the moisture to escape. The goal of this study was to assess the impact of such liners.

**METHODS:**

A sample group of 13 patients with unilateral transtibial amputation, who wore a perforated liner (PL) as part of their current prescription, was compared to 20 control patients who wore non-perforated liners (NPL). During their routine appointments, they completed a survey of scientifically validated outcome measures relating to their limb health, pain and the impact on daily life over a 12-month period.

**RESULTS:**

Patients using the PL had healthier residual limbs, reporting higher scores on questions relating to limb health, experiencing fewer skin issues (p<0.001) and estimating a 61.8% lower rating in perceived sweat (p=0.004). Perhaps consequentially, there was a lower incidence of residual (p=0.012) and phantom (p=0.001) limb pain when compared to the control group. The prevalence of individual issues affecting the residual limbs of PL users was also lower. Of the issues that remained, only 23% were attributed to sweating in PL users, compared to 49% for the NPL group (p=0.066). PL users missed fewer days of work in the year (2.4 vs 11.6, p=0.267) and were also limited on fewer days (1.4 vs 75.4, p=0.009).

**CONCLUSION:**

The use of perforated liners shows much promise within prosthetic care, significantly improving the health of the residual limb. The observed effects on perceived sweat reduction, residual skin health, pain levels and patient limitation suggest that perforated liners are highly beneficial to patients.

## INTRODUCTION

Excessive sweating at the residual limb affects the quality of life of up to 70% of people with amputation and is one of the most common complaints reported by prosthetic users.^1–3^ Indeed over 53% of lower limb amputees reported discomfort due to heat and/or perspiration^4^ while 66% felt that sweating impacted on their daily activities.^5^

Comparatively, only 2.9% of the general population have been medically diagnosed as suffering from excessive sweating, or “hyperhidrosis”.^6^

Sweating forms an effective way for reducing temperature and is an essential component of the body’s cooling process.^7^ When the skin is disrupted in some way, the body adapts in order to achieve the same cooling benefits and it is this adaptation that can prove problematic within prosthetics. People with lower limb amputation generally exert higher levels of energy during ambulation compared to able-bodied people. For unilateral transtibial amputees, this was around 16%,^8^ while with bilateral transtibial amputations, it is closer to 40%.^9^ Consequentially, body temperature rises and the body reacts accordingly. A transtibial amputation also reduces the skin’s surface area by around 10-15%,^10^ so in order to achieve the same cooling effect, the rate of sweat production from the remaining surface must increase proportionally. The combination of these factors means that people with amputation may produce more sweat.

Prosthetic liners increase layers above the skin, offering protection and comfort to sensitive areas, but the materials are not breathable and act as insulators.^11^ This exacerbates the rise in temperature and traps the resultant sweat on the skin’s surface. Sustained exposure to moisture has a detrimental effect on the skin,^12^ leaving it more susceptible to injury. Skin disorders also become more problematic within the amputee population due to scar tissue and the high prevalence of vascular disease.^13^ This compromises blood flow, reducing healing capabilities and making the skin vulnerable.^14,15^ Renshaw found that skin previously wetted with water was more likely to blister than when it was dry.^16^ In addition, Hurkmans et al. found that sweat accumulation contributed towards skin irritation, perhaps even more so than infection.^17^ Perhaps unsurprisingly then, skin disorders are prevalent in this vulnerable population^18^ with wound infection rates ranging between 13-40%^19^ following amputation and residual limb pressure ulcers causing the majority of re-amputations.^20–22^

Various approaches have been trialled to improve excessive perspiration and heat,^4^ from the use of commercial or prescription anti-perspirants^23^ to more extreme options such as Botulinum Toxin injections.^24,25^ More prosthetic-specific solutions have also been tried and evaluated.^26^ Wernke et al. investigated the SmartTemp liner^27^ (Ohio WillowWood, Mt Sterling, OH, USA), which uses Phase Change Material in order to store and release heat energy. The liner effectively reduced the initial temperature of the residual limb and therefore the volume of sweat produced. However, these materials have a limit on the amount of cooling they provide^28^ and in another study^29^ thermally conductive silicone did not result in a significant improvement in climate control over plain silicone liners. More recently, a liner with perforations has been developed (Silcare Breathe, Blatchford, Hampshire, UK) allowing moisture to escape and keeping the residual limb dry.^30^ These liners reduced the prevalence of sweat remaining on the skin in all participants during trials^30^ and in published case studies.^31^ Evidence has suggested they are effective in the management of wounds and beneficial to residuum skin health, especially when used in combination with elevated vacuum.^31^

This study sought to determine the efficacy of perforated prosthetic liners for the purpose of sweat management, investigating what impact, if any, this made on patient outcomes.

## METHODOLOGY

### Evaluated technology

**Figure 1:**
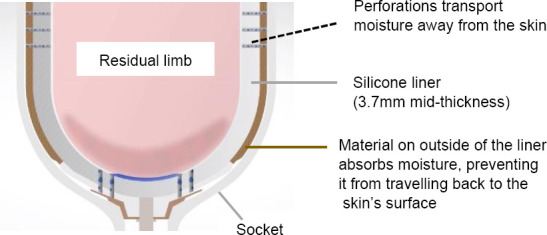
A cross-sectional image of the Silcare Breathe liner (Blatchford).^I, II^ Moisture is transported away from the skin through perforations located along the sides and at the distal end of the liner.

This study examines the effects of a perforated liner (PL - Silcare Breathe, Blatchford Products Ltd., Hampshire, UK)^I, II^ on patient outcome measures and residual limb health. It differs from previous silicone designs because it incorporates perforations along the length, and at the distal end, with the intention of improving skin interface microclimate control and hygiene of the residual limb (Figure 1).

These perforations allow moisture produced by the body to be transported to the outside of the liner, so that it doesn’t remain on the surface of the skin. Moisture is then readily absorbed by the fabric on the outside of the liner, rather than by the silicone, so that it does not migrate back towards the skin and the liner can be more easily cleaned.

### Participants

Suitable candidates (n=41) for the study were identified and approached by their prosthetists during routine appointments at Northern General Hospital in Sheffield. The cohort was then divided into PL users and a control group who wore conventional non-perforated liners (NPL). Participants had to have a transtibial amputation and were required to have been using their current prescription for a minimum of 12 months. All participants were established prosthesis users with a mobility level of K2 or above and were able to read and write in English, with sufficient cognitive ability.

All patients provided informed, written consent. The study was approved by the Clinical Effectiveness Unit at Sheffield Teaching Hospitals. No interventional actions were taken and the ethics of the study conformed with the World Health Organisation Declaration of Helsinki.^32^

### Data collection and measures

All participants completed a survey of scientifically validated outcome measures during routine appointments. The surveys were comprised of selected sections of the Patient Evaluation Questionnaire^33^ (PEQ); a clinically validated patient-reported outcome measure that has been used successfully to analyse the use of prosthetic liners in previous studies.^34–36^ This questionnaire is organised into independent functional domain subsections,^37^ where the patient indicates a score out of 100 using a visual analogue scale (VAS) for each individual question. Every question relates to the previous four weeks and the overall average gives the score for the subset.

For this study, the residual limb health subset was used, made up of questions about sweat inside the liner, smelliness, swelling, rashes, ingrown hairs and blisters. As well, individual PEQ questions relating to frequency, intensity and ‘bothersomeness’ of residual and phantom limb pain were included. Following PEQ scoring, lower scores indicated worse symptoms (e.g. 0 = extremely intense, 100 = extremely mild). For frequency of pain, a seven-point multiple choice format was used, rather than the visual analogue scale. These choices were “Never”, “Only once or twice”, “A few times (about once/week)”, “Fairly often (2-3 times/week)”, “Very often (4-6 times/week)”, “Several times every day” and “All the time or almost all the time”. In order to allow a quantifiable comparison between groups, these responses were assigned a score from 1 (least frequent) to 7 (most frequent), from which the mean and SD were calculated.

In addition, questions specifically designed for this study were included in the surveys which related to the previous 12 months. These enquired about excessive sweating, number of socket adjustments required, what issues the patient experienced on their residual limb and the number of days work that had been missed or limited because of issues caused by sweating.

Study group participants also filled out the survey retrospectively, with regards to their previous prescription. Due to the retrospective nature of these responses, it was thought that results might be unreliable and affected by bias. While the retrospective responses were comparable to those of the control group, the principal comparison reported in this work is between the study group’s current responses and responses from the control group.

### Data processing and analysis

Residual limb issues were categorised into conditions and the frequency of each was recorded as a percentage of the population so that an objective comparison could be made. Participants were asked to estimate what percentage of their issues they attributed directly to sweat. This figure was then multiplied by the total number of issues each participant suffered from, to calculate the number of issues that each participant attributed to sweating.

For all VAS and numerical responses, the mean and standard deviation for each outcome measure were found and used for comparisons. Data were tested for normality using the Shapiro-Wilk test. Homogeneity of variance was assessed using F tests for normal data and a Fligner-Killeen test in cases where the data were not normal. Dependent on the outcome, comparisons of means were made using t-tests, Wilcoxon tests or Kruskal-Wallis tests. For ordinal or nominal data (e.g. frequency of pain and prevalence of issues), comparisons were made using a chi-squared analysis. For all tests, p<0.05 indicated significance.

## RESULTS

### Demographics

Of the overall cohort (n=41), 21 were identified as PL users. Out of these, eight patients did not use the liner consistently, or discontinued use, and were excluded. Of the remaining 13 participants, ten were male and three were female (age: 49 (SD=10) years; weight: 96 (SD=26) kg. Nine had used silicone liners in their previous prescription and four used conventional pelite liners in conjunction with a suspension sleeve; all now wore perforated silicone liners with either suction or pinlock suspension (Table 1).

**Table 1:** Study group participant demographics.

	Gender	Age (years)	Weight (kg)	Aetiology	Previous prescription (suspension)	Current prescription (suspension)
**PL01**	M	57	92.5	Unknown	Pelite liner and silo sheath (sleeve)	Blatchford Silcare Breathe Cushion (suction)
**PL02**	F	37	103	Infection	Pelite liner and silipos sock (sleeve)	Blatchford Silcare Breathe (pinlock)
**PL03**	M	54	112	Trauma	Össur liner (pinlock)	Blatchford Silcare Breathe (pinlock)
**PL04**	M	41	100.6	Trauma	Blatchford liner (pinlock)	Blatchford Silcare Breathe (pinlock)
**PL05**	F	55	77	Congenital	Össur liner (pinlock)	Blatchford Silcare Breathe (pinlock)
**PL06**	M	35	81.4	Infection	Pelite liner (sleeve)	Blatchford Silcare Breathe (pinlock)
**PL07**	M	62	99.3	Unknown	Össur liner (pinlock)	Blatchford Silcare Breathe (pinlock)
**PL08**	M	57	82.1	Vascular	Össur liner (pinlock)	Blatchford Silcare Breathe (pinlock)
**PL09**	F	38	79.2	Trauma	Blatchford cushioned liner (suction)	Blatchford Silcare Breathe (pinlock)
**PL10**	M	46	88.2	Trauma	Alps liner (pinlock)	Blatchford Silcare Breathe (pinlock)
**PL11**	M	59	104.8	Trauma	Alps liner (pinlock)	Blatchford Silcare Breathe (pinlock)
**PL12**	M	59	169.6	Vascular	Pelite liner (sleeve)	Blatchford Silcare Breathe Cushion (suction)
**PL13**	M	41	62.9	Pain Management	Blatchford cushioned liner (suction)	Blatchford Silcare Breathe (pinlock)
**Mean**	-	49.3	96.4	-	-	-
**SD**	-	9.8	25.9	-	-	-

The control group was made up of the remaining 20 participants, 16 male and four female (age: 56 (SD=15) years; weight: 90 (SD=22) kg). All of them wore silicone prosthetic liners, using either suction or pinlock suspension, from various distributors (Table 2).

**Table 2:** Control group participant demographics.

	Gender	Age (y)	Weight (kg)	Aetiology	Previous prescription (suspension)
**NPL01**	F	49	80.0	Infection	Össur liner (pinlock)
**NPL02**	M	76	83.5	Trauma	Össur liner (pinlock)
**NPL03**	M	67	91.8	Trauma	Össur liner (pinlock)
**NPL04**	M	66	106.4	Infection	Alps liner (pinlock)
**NPL05**	M	30	69.3	Trauma	Össur liner (pinlock)
**NPL06**	M	70	79.6	Trauma	Össur liner (pinlock)
**NPL07**	M	31	83.6	Infection	Alps liner (pinlock)
**NPL08**	M	81	71.8	Vascular	Blatchford liner (pinlock)
**NPL09**	M	59	94.6	Trauma	Alps liner (pinlock)
**NPL10**	F	57	84.6	Trauma	Össur liner (pinlock)
**NPL11**	M	70	74.4	Vascular	Össur liner (pinlock)
**NPL12**	M	56	97.6	Trauma	Blatchford liner (pinlock)
**NPL13**	M	47	85.0	Trauma	Alps liner (pinlock)
**NPL14**	M	63	97.5	Unknown	Ottobock cushioned liner (suction)
**NPL15**	M	48	166.0	Unknown	Alps liner (pinlock)
**NPL16**	M	47	67.0	Vascular	Alps liner (pinlock)
**NPL17**	M	52	103.0	Unknown	Össur cushioned liner (suction)
**NPL18**	F	31	70.0	Trauma	Blatchford liner (pinlock)
**NPL19**	M	79	89.0	Trauma	Ottobock custom silicone liner (pinlock)
**NPL20**	F	47	116.0	Trauma	Össur liner (pinlock)
**Mean**	-	56.3	90.5	-	-
**SD**	-	15.4	22.1	-	-

### Residual limb health and pain

**Figure 2:**
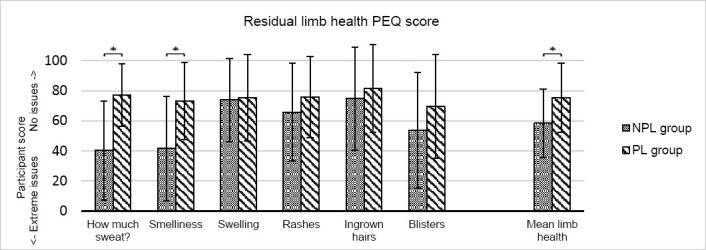
Residual limb health PEQ subset score for the NPL control group (grey) and the PL group (green). The error bars indicate ± one standard deviation from the mean. Asterisks (*) indicate a statistically significant change (p<0.05).

The mean residual limb health score for the PL group (75.0 (SD=22.9)) was significantly greater than that of the NPL group (58.4 (SD=22.7); p=0.046). For each of the six questions that constitute this subset, mean responses were higher for the PL group, indicating that problems were less prevalent, and that the limb was healthier. For two questions – the amount of sweat in their liner (p=0.004) and how smelly their limb was (p=0.012) – the difference in response reached statistical significance (Figure 2). Indeed, if the scale is inverted (so that a higher number indicates a higher quantity of sweat) PL users reported 61.8% less sweat remaining on the limb than the control group (p=0.004).

When asked a binary yes/no question about whether they perceived the amount they sweat to be excessive (Table 3), 85% of the control group answered yes. In the study group, this was significantly less (p<0.001) with only 15% reporting excessive sweating. It is also worth noting that these participants added the caveat that this occurred only very rarely.

**Table 3:** Results of additional questions included within survey. P values in bold text indicate significance (p<0.05).

During the past 12 months…	PL group (Mean (SD))	NPL group (Mean (SD))	p value
Do you currently suffer from excessive sweating?	Yes: 2 (15.4%) No: 11 (84.6%)	Yes: 17 (85.0%) No: 3 (15.0%)	**<0.001**
Approximately, how many socket adjustment appointments have you required?	1.8 (1.9)	2 (1.4)	0.610
What % of your residual limb issues (skin/tissue breakdown) would you attribute to sweating?	22.7 (33.2)	49.0 (39.5)	0.066
Have you taken any days off work, or were housebound, for skin issues? If so, how many days?	N = 12 2.4 (6.0)	N = 16 11.6 (21.9)	0.267
Have you limited the use of your prosthesis and activities due to discomfort caused by sweating? If so, how many days?	N = 12 1.4 (2.9)	N = 17 75.4 (130.6)	**0.009**

PL users reported less intense residual limb pain (66.3 (SD=32.0)) and less intense phantom limb pain (48.6 (SD=35.4)) than the NPL control group (38.5 (SD=29.9)) and 37.2 (SD=37.0) respectively). While neither of these changes reached statistical significance (p=0.071 and p=0.360 respectively), how “bothersome” participants found pain in their residual limb did, with the PL group reporting that it was less bothersome (68.5 (SD=30.9)) than the NPL group (38.8 (SD=32.0), p=0.045).

**Figure 3:**
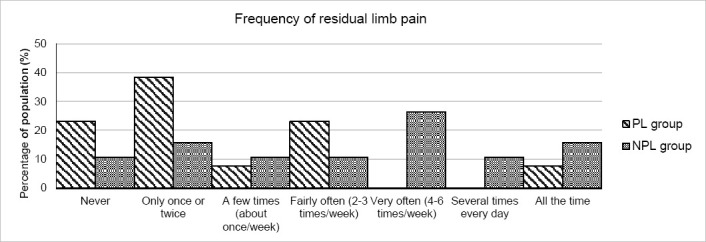
PEQ pain subset results for the frequency of the residual limb pain experienced by both PL (striped) and NPL (grey) groups. A skew to the left indicates less pain. The difference between the group was significant (p<0.05) with PL users experiencing less pain.

**Figure 4:**
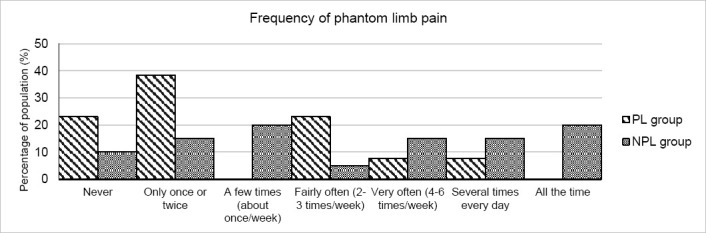
PEQ pain subset results for the frequency of phantom limb pain experienced by both PL (striped) and NPL (grey) groups. A skew to the left indicates less pain. The difference between groups was significant (p<0.05) with PL users experiencing less pain.

Significant differences were observed for the PL group, compared to the NPL group, in the frequency of residual limb pain (2.7 (SD=1.7) and 4.2 (SD=1.9), respectively; p=0.032) and the frequency of phantom limb pain (2.8 (SD=1.6) and 4.3 (SD=2.1), respectively; p=0.042). These results are shown in Figure 3 and Figure 4. A clear skew towards lower frequencies in the PL group here, indicates fewer occurrences of pain.

### Issues at the residuum

The mean number of residual limb issues reported was significantly higher in the NPL control group (2.8 (SD=1.5)) than the PL group (1.2 (SD=1.0), p<0.001).

**Figure 5:**
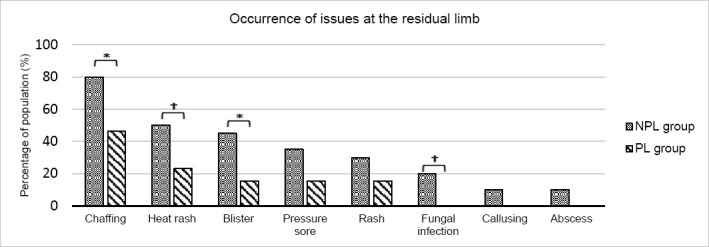
Percentage of the group populations that were affected by individual issues at the residual limb. The NPL group are indicated in grey and the PL group in green. Asterisks (*) indicate statistically significant results (p<0.05) while obelisks (†) indicate results that approached significance (0.05<p<0.10).

Issues that were reported by both groups were chaffing, blisters, rashes, heat rash and pressure sores, with chaffing being the most prevalent issue in both groups (Figure 5). The frequency of all issues was lower in the PL group; the percentage of the population affected by chaffing differed significantly from 80% in the control group, to 46% in the PL group (p=0.002).

The occurrence of blisters also changed significantly from 45% in the control group to 15% in the PL group (p=0.032); and differences in heat rashes (50% vs 23%) and fungal infection (20% vs 0%) approached significance (p=0.052 and 0.071 respectively). Although not significant, the occurrence of pressure sores changed from 35% in the control group to 15% of the PL group (p=0.138). Least prevalent issues within the control group (fungal infection, callusing, abscess) were not seen at all in the PL group.

**Figure 6:**
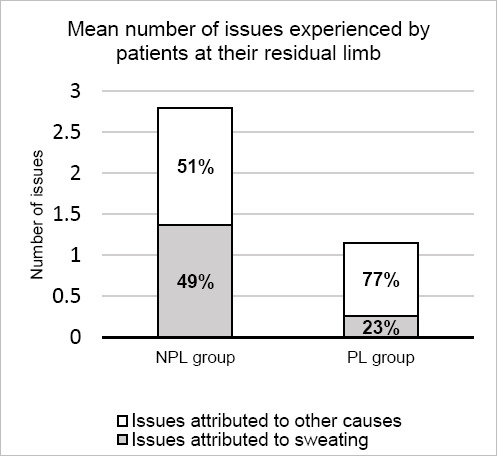
The mean number of issues experienced by patients at their residual limb, and the percentage of those issues that were attributed to sweating (grey) or to other causes (green). Exact percentages are indicated within bars.

When patients were asked to rate how many of their issues they attributed directly to sweating, the percentage was lower for the PL group (Table 3). The control group estimated that on average, 49% of all issues were due to sweating, whereas the PL group attributed only 23% to sweat (Figure 6).

This result did not reach statistical significance (p=0.066) due to the high variability between patients. When using the percentages to calculate the number of issues attributed to sweat however, the control group subjects averaged 1.7 (SD=1.7), while in the study group this was 0.3 (SD=0.5); a result that was significant (p=0.007).

The number of workdays missed was lower in the PL group with a mean of 2.4 (SD=6.0) days taken off work due to issues compared to 11.6 (SD=21.9) in the control group (p=0.267, Table 3). The number of days limited by issues surrounding the prosthesis was significantly less, with the PL group limited a mean of 1.4 (SD=2.9) days and the control group 75.4 (SD=130.6) days (p=0.009).

## DISCUSSION

This report investigated the clinical consequences of perforated prosthetic liners based on the feedback provided by study participants and a comparable control group. While patient-reported outcome measures can be subjective, large trends in the data can imply significant changes in patient outcome over a wider population. The results suggest perforated liner users have significantly more successful patient outcomes and experiences through better residual limb health and less frequent outbursts of pain.

Overall, the study group showed better PEQ residual limb health scores (Figure 2). Within this subset, the score for every question was higher, indicating an increase in health and therefore a reduction of each adverse factor. Significant changes were seen in the amount of sweat present on the limb as well as in the limb’s odour. Given that malodour is a direct result of the bacterial secretions following a transformation of the compounds found in sweat,^38^ it seems logical that a decrease in odour would be attributable to a decrease in the amount of sweat inside the liner. The design of the perforated liner is such that once the moisture migrates to the outside of the liner, it is absorbed by the fabric lining. To this end, the odour is more likely to develop on the outside of the liner, and can be easily washed, instead of impregnating into the inner silicone where it lingers.

For clarification, it is worth highlighting that this study used patient reported outcome measures and so reported the patient’s perception of whether they sweat to excess and how much remained on their limb. This study cannot comment on whether the type of prosthetic liner used affects the actual quantity of sweat produced. However, sweat being transported away from the skin is likely to have positively affected the amount of sweat perceived by participants using the perforated liner, as well as contributing towards a healthier environment for the residual limb.

There were notable differences in scores relating to rashes and blisters within the PEQ subset (Figure 2). Again, these are largely foreseeable. Rashes can be caused by infection, a known risk factor in the bacteria-rich environment caused by trapped sweat, but they can also be the result of friction.^39^ Additional lubrication causes movement at the socket^11^ and moisture increases the coefficient of friction between the skin and materials in contact with it,^40^ imposing greater risk of tissue breakdown such as blisters. It follows then, that removing sweat from the skin’s surface might reduce the amount of irritation caused by movement at the residuum-socket interface, therefore decreasing the likelihood of rashes and blisters. Scores regarding swelling and ingrown hairs also showed less prevalence in the PL group, but the difference was small, suggesting that climate control affected these to a lesser extent. However, previous studies^41^ have drawn links between water exposure and resulting inflammation of the skin, so it might be of interest to explore further the relationship of climate control to individual factors and conditions that affect the residual limb.

For the PEQ questions relating to pain, both residual and phantom limb pain were significantly less frequent in the PL group (Figure 3 and Figure 4). The difference in residual limb pain might have been predicted due to the discomfort caused by physical skin conditions which were less prevalent. However, the cause of phantom limb pain, which occurs after up to 80% of amputations,^42^ is still a subject of speculation and continued investigations are needed.^43^ Larbig et al.^44^ surmised that, along with other factors, physical pain following amputation was a risk factor for more intense phantom limb sensations, maybe providing some explanation of the reduction reported in this study. In addition, Fuchs et al.^45^ measured significantly higher intensities of phantom limb pain in upper limb amputees with lower heat pain thresholds. Although the difference in the intensity of phantom limb pain was not significant in this study, the suggested link between that and the temperature of the limb is an interesting one and may warrant further study.

The proportional prevalence of specific skin conditions was consistent with that found in previous studies^46,47^ across both patient groups, however the frequency of issues was less with the use of the perforated liner. The reduction in the average number of skin issues in the study group reinforces the findings from a previous study.^31^ Not only was the number of issues present at the residual limb less, but the same was true with the occurrence of more severe conditions. As previously discussed, less perspiration on the skin’s surface might result in lower rates of infection and irritation; but it may also contribute to a reduction in calluses and pressure sores by reducing movement of the liner. The formation of ulcers is included within the category of pressure sores, which are arguably one of the most serious issues that can occur, linked to vascular disease for which a large number of lower limb amputations are attributable.^13^

Historically, complications with the skin of the residual limb limit prosthetic use, interfering not only with an established patient’s ability to ambulate, but also with the rehabilitation process of primary amputees. This limits participation, affecting quality of life^48,49^ and patient wellbeing,^46^ leading to negative implications for the physical, psychological and emotional condition of the patient. Indeed primary patients who remained non-ambulatory for over 6 months were shown to have a much higher likelihood of developing complications,^50^ with several studies^51–53^ finding early prosthetic use in primary amputees had real benefits not just for the mental health of patients but for the subsequent outcome measures such as treatment compliance and prosthetic use.

As such, it is important to minimise the time that patients spend without a prosthesis. Gallagher et al.^54^ found one of the top three factors affecting amputee participation was climate. Furthermore, pain in the residual and phantom limbs has been found to influence a patient’s ability of returning to work following amputation.^55^ Within this study, PL users had fewer days of limited prosthetic use and a lower number of workdays missed. This, combined with the perception of improved climate control and limb health reported, would indicate an improvement in patient outcomes. It is worth noting, that while these results accounted for the active employment status of participants, the type of employment was not considered. The type of work may have affected the ability of the individuals to work given socket discomfort and therefore the number of days missed.

The primary aim of this study was to identify the health effects of different liners on the residual limb. However, the implications that patient health and reduction in rehabilitation times have in terms of health and economic cost is well documented.^56,57^ In addition, days of work that are either missed by patients or are limited, affect not only the individual’s salary but can increase vulnerability in the face of redundancy or promotion opportunities.^58^ The health economic benefits require further investigation, however, the possible larger scale implications of this treatment pathway only increase the clinical significance of this study.

Participants were selected by clinicians using inclusion criteria and asked to take part in the study during normal clinical visits. As this was not a blind recruitment process, it may have introduced a bias. The control group were selected at random from established patients attending the clinic, however the study group were selected because of their specific prescription. Patients are likely to be using this prescription because of higher activity levels or past problems related to sweating; equally the control group could have a large range in activity levels. All participants were activity level K2 or above, however the activities of each patient were not recorded. Although the data are likely to show a bias against perforated liners, potentially making the effects of this intervention more powerful, the lack of activity data is considered a large limitation of this study.

Participants were asked a binary question about whether or not they considered their sweating excessive. The percentage of participants who answered yes was significantly less in the study group compared to the control group (p<0.001, Table 3). This is useful in that it provides an awareness of patient experience and satisfaction, but it does not show any variation between the patients that sweat to excess. Although still subjective, the nature of the PEQ requires participants to quantify the amount they think they sweat, thereby accounting for participants who felt they fell somewhere in the middle. This question gave a similar result, with the study group reporting significantly higher scores in the PEQ questions relating to the quantity of sweat present (p=0.004) on the residual limb. Both questions essentially give very similar data, but it could be argued that in this case, the PEQ provides more information.

Although many patients with lower limb amputations suffer from it, vascular disease is the aetiology of surprisingly few participants within this study. The slower healing capabilities of this demographic, means these patients are more likely to suffer from skin conditions^18^ and so the lack of vascular participants might mean the study is not representative of the overall amputee population. However, the study and control groups are comparable in this respect, and there is still a significant difference seen in the prevalence and type of skin conditions.

One aspect of the study population that is of note, is the complete absence of one or more skin conditions on the residual limb in the study group (Figure 5). This is a significant improvement; however, given the small sample size, the result is likely a false positive. If the number of participants had been larger, it is probable that these issues would have occurred, at least minimally, in both groups.

There was a high variability in the percentage of issues that patients attributed to sweating, meaning that the result was not considered significant. This variation came from the balance between the number of issues caused by sweating compared with the total number of issues each participant experienced. For example, a patient may only have one issue, but it may be 100% the result of sweating. This variation calls into question the validity of the result. By multiplying the individual’s total number of issues by the percentage that they estimated were caused by sweat, the number of sweat resultant issues that each patient suffered from could be calculated. This showed a reduction in sweat related issues which did prove significant (p=0.007, Table 3) and so it must be noted that although the percentages may not be significant on their own, when applied to the data on issues suffered by the individual, the result is valid (Figure 6).

It is possible that some patients may experience iatrogenic affects from the perforations, however this was not reported by any of the participants in the study. Additional factors that could affect the outcome measures of this study included liner material and thickness, suspension type and socket comfort. The perforated liner used in this study is 3.7mm thick at its mid-point and so falls within the industry standard range (3mm to 6mm). Although manufacturers use different material compositions, Ali et al. found that the was no significant difference between the sweat complaints of patients using liners of different material and varying suspension method.^59^ Nevertheless, it should be noted that all participants used either pinlock or suction suspension systems. Socket comfort scores were not collected in this study, however the number of socket adjustments needed in the 12-month period was recorded (Table 3). Socket adjustments are often required if there is discomfort and so might indicate a level of socket comfort. The number of adjustments needed was comparable between groups, so this factor is unlikely to have had any significant impact on the results of this study.

## CONCLUSION

This study shows promising results for the use of perforated liners within prosthetic care, with significant differences being observed between the two patient groups. Excessive sweating impacts many aspects of the patient’s life beyond just their physical health; resulting issues preventing prosthetic use and limiting patients’ mobility, in turn affecting their daily lives and causing frustration. Perforated liners help to manage excessive sweat levels. Improvements in patient health and the implications that this may have on quality of life often inform prescription guidelines, therefore technology that can positively impact these factors may prove highly beneficial.

## DECLARATION OF CONFLICTING INTERESTS

The authors are full time employees of Blatchford, the manufacturer of the prosthetic liner evaluated in this study.

## AUTHOR CONTRIBUTION

**Katherine C. Davies:** Data analysis, writing original, review and editing

**Mike McGrath:** Conceptualisation, data analysis, writing original, review and editing

**Zoe Savage:** Conceptualisation, data collection

**Alison Stenson:** Conceptualisation, data collection

**David Moser:** Review and editing

**Saeed Zahedi:** Review and editing

## SOURCES OF SUPPORT

Authors are employees of Blatchford Products Ltd.

## ETHICAL APPROVAL

All patients provided informed, written consent. The study was approved by the Clinical Effectiveness Unit at Sheffield Teaching Hospitals. No interventional actions were taken and the ethics of the study conformed with the World Health Organisation Declaration of Helsinki.

## MANUFACTURERS’ DOCUMENTATION

I- https://www.blatchford.co.uk/products/silcare-breathe-cushionliner/

II- https://www.blatchford.co.uk/products/silcare-breathe-lockingliner/
